# Role of IL-6/JAK/STAT pathway in inducing vascular insulin resistance

**DOI:** 10.1186/1755-8166-7-S1-P96

**Published:** 2014-01-21

**Authors:** Aswath Balakrishnan, Kapaettu Satyamoorthy, Manjunath B Joshi

**Affiliations:** 1 Division of Biotechnology, School of Life Sciences, Manipal University, Manipal, India

## Introduction

Insulin resistance is a hall mark of metabolic disorders. Studies have demonstrated that inflammatory regulator interleukin-6 (IL-6) plays an important role in disruption of IR/Akt/eNOS signaling pathway resulting vascular insulin resistance. Accumulating evidences suggests a significant role of epigenetic mechanisms such as DNA methylation in progression of metabolic disorders. Hence the present study aimed to understand the role of epigenetic mechanisms involved during IL-6 induced vascular insulin resistance and its consequences in cardiovascular diseases.

## Materials and methods

Human umbilical vein endothelial cells (HUVEC) and Human dermal microvascular endothelial cells (HDMEC) were used for this study. Endothelial cells were treated in presence or absence IL-6 (20ng/ml) for 36 hours and followed by insulin (100nM) stimulation for 15 minutes. Levels of phosphorylated- and total Akt served as readout for insulin resistance. To investigate changes in DNA methylation, cells were treated with or without neutrophil conditioned medium (NCM) as a physiological source of inflammation or IL-6 for 36 hours. Genomic DNA was processed for HPLC analysis for methyl cytosine content and cell lysates were analyzed for DNMT1 (DNA (cytosine-5)-methyltransferase 1) and DNMT3A (DNA (cytosine-5)-methyltransferase 3A) levels using immunoblotting.

## Results

Endothelial cells stimulated with insulin exhibited an increase in phosphorylation of Akt ^ser 473^ in serum free conditions but such insulin response was not observed in cells treated with IL-6, suggesting chronic exposure of endothelial cells to IL-6 leads to insulin resistance. HPLC analysis for global DNA methylation resulted in decreased levels of methyl cytosine in cells treated with pro-inflammatory molecules (both by NCM and IL6) as compared to 3.2% in untreated control to 2% in treated. Kinetic studies depicted a transient increase DNA methylation at 24 hours which was followed by steep decrease at 36 hours. Subsequently, analysis in cells treated with IL-6 showed a significant decrease in DNMT1 levels but not in DNMT3A. Other pro-inflammatory marker such as TNF-α did not exhibit such changes. Interestingly we also observed Akt phsophorylation refelcetd DNMT1 changes suggesting plausible role of PI3K/Akt signaling axis in regulation of DNMT1 expression.

## Conclusion

Taken together our study suggests that IL-6 induces vascular insulin resistance and involvement of epigenetic changes.

